# Acknowledgements

**DOI:** 10.1186/s12879-026-13216-7

**Published:** 2026-06-11

**Authors:** Igor Toskin, David Lewis

**Affiliations:** 1 World Health Organization, Geneva, Switzerland; 2 Westmead Clinical School, University of Sydney, Westmead, Australia


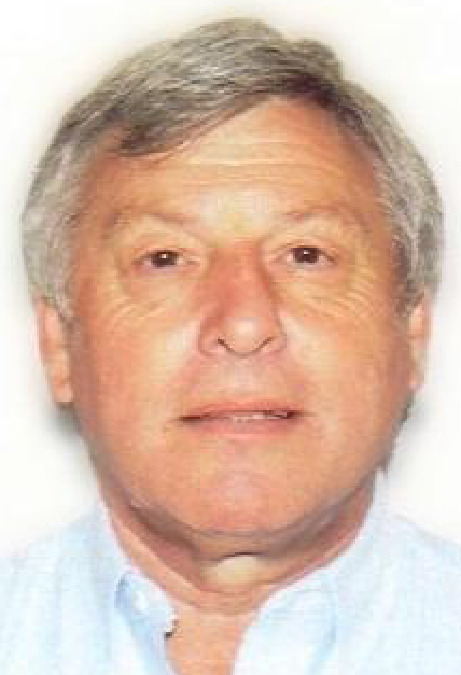


This Special Issue of *BMC Infectious Diseases* summarises the results from an independent multi-country (15 countries, 30 sites, over 17,000 patients) clinic-based and clinic-utility evaluation of point-of-care tests (POCT) for sexually transmitted infections (STIs) led by the World Health Organization (WHO).

Dr. Ronald Campbell Ballard (1946-2026) provided extensive advice to the conceptual design of the PRoSPeRo Project was involved in this Special Issue as a Supplement Editor.

Throughout his remarkable career, Dr. Ballard occupied several senior positions in the field of STI control and prevention including Director of the National Reference Centre for Sexually Transmitted Diseases (STD) at the South African Institute for Medical Research (SAIMR) and Associate Professor of Medical Microbiology and Infectious Diseases at the Medical School of the University of Witwatersrand, Johannesburg, South Africa. Later, he was appointed Chief of the STD Laboratory Branch at the Centers for Disease Control and Prevention (CDC), in Atlanta GA, USA. In 2014, Dr. Ballard was subsequently appointed Associate Director for Laboratory Science, in a newly-formed Center for Global Health at CDC, in Atlanta.

Dr. Ballard authored more than 300 papers presented at international and local meetings and congresses on various subjects related to ocular and genital tract infections and published over 150 publications in peer reviewed scientific journals.

Dr. Ballard was an internationally recognized investigator and expert in STIs, microbiology and laboratory medicine. For many years, he continued to lead efforts to develop new diagnostic technologies for STIs, including point-of-care tests, and new interventions to improve access to STI and sexual health services. In addition, Dr. Ballard led several impactful research projects and initiatives that aim to improve STI control and prevention as well as strengthen laboratory expertise and research capacity in several countries ranging from basic research to clinical trials.

Dr. Ballardwas a member of several WHO Expert Groups, including the WHO Committee on prevention of blindness, WHO/UNAIDS committees on syndromic management of STDs, and the WHO Director-General’s Advisory Panel on Sexually Transmitted Infections.

WHO would like to acknowledge Dr. Ballard for his outstanding contribution to international efforts to strengthen STI control and prevention at national and international levels during his lifetime, and for his notable contribution to the development of this Special Issue of the BMC Infectious Diseases entitled “Point-of-care Testing for Sexually Transmitted Infections: results of an independent multi-country clinic-based and clinic-utility evaluation of STI diagnostics (PRoSPeRo project).”

